# Vaccines for neglected diseases: challenges and opportunities

**Published:** 2011-01-10

**Authors:** Allan Saul

**Affiliations:** 1 Novartis Vaccines Institute for Global Health, 53100 Siena, Italy

## 

Infectious diseases exert a major burden of disease in developing countries with 99% of the global burden of infectious diseases, as measured by DALYs, in low and middle income countries. While better use of existing vaccines would make an appreciable difference, the greatest burden is caused by diseases for which we currently have no vaccines. The picture, especially in children, is dominated by diarrheal and respiratory diseases. Paradoxically these diseases have relatively low priority for funding in absolute terms, and especially in relationship to the burden of disease. Thus, new vaccines for these neglected diseases need both innovative scientific solutions and innovative development schemes involving scientific institutes, public financing and industrial input. The industrial input is critical: not only will vaccine manufacture require an industrial partner, but the knowledge to efficiently undertake the technical and clinical development leading to vaccine production largely resides in industry. A potentially important development in this area has been the recent formation of Industry Linked Vaccine Institutes: For example, the Novartis Vaccines Institute for Global Health and the Hilleman Laboratories. These are an important conduit for applying industrial know how for developing commercial vaccines to the pressing need for vaccines for neglected diseases of developing countries.

